# Supplementary material for: Protection Efficacy of Monoclonal Antibodies Targeting Different Regions of Specific SzM Protein from Swine-Isolated Streptococcus equi ssp. zooepidemicus Strains
Source: Microbiol Spectr. 2022 Oct 18;10(6):e01742-22. doi: 10.1128/spectrum.01742-22 (PMC9769693; doi:10.1128/spectrum.01742-22)
Supplement: Supplemental file 1 — Fig. S1-S5, Table S1. Download spectrum.01742-22-s0001.pdf, PDF file, 0.6 MB [file spectrum.01742-22-s0001.pdf]

Fig S1

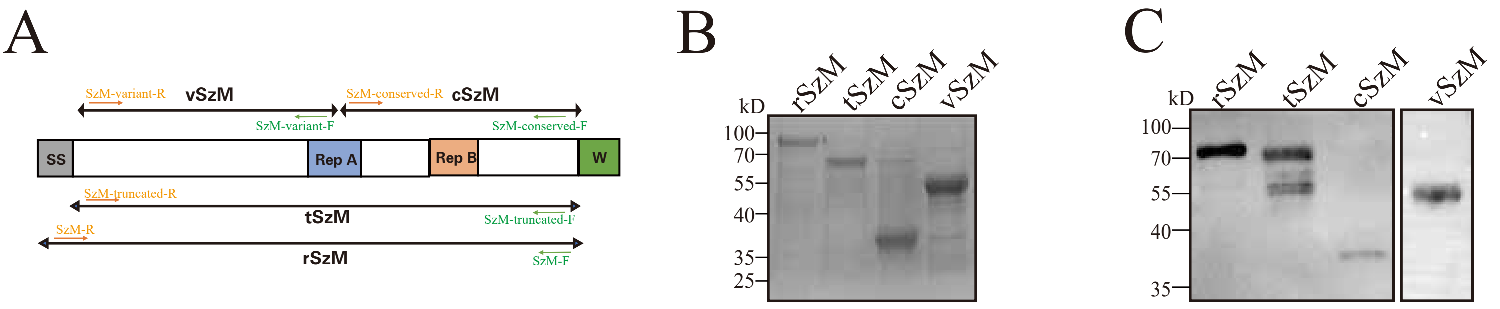

Fig S2

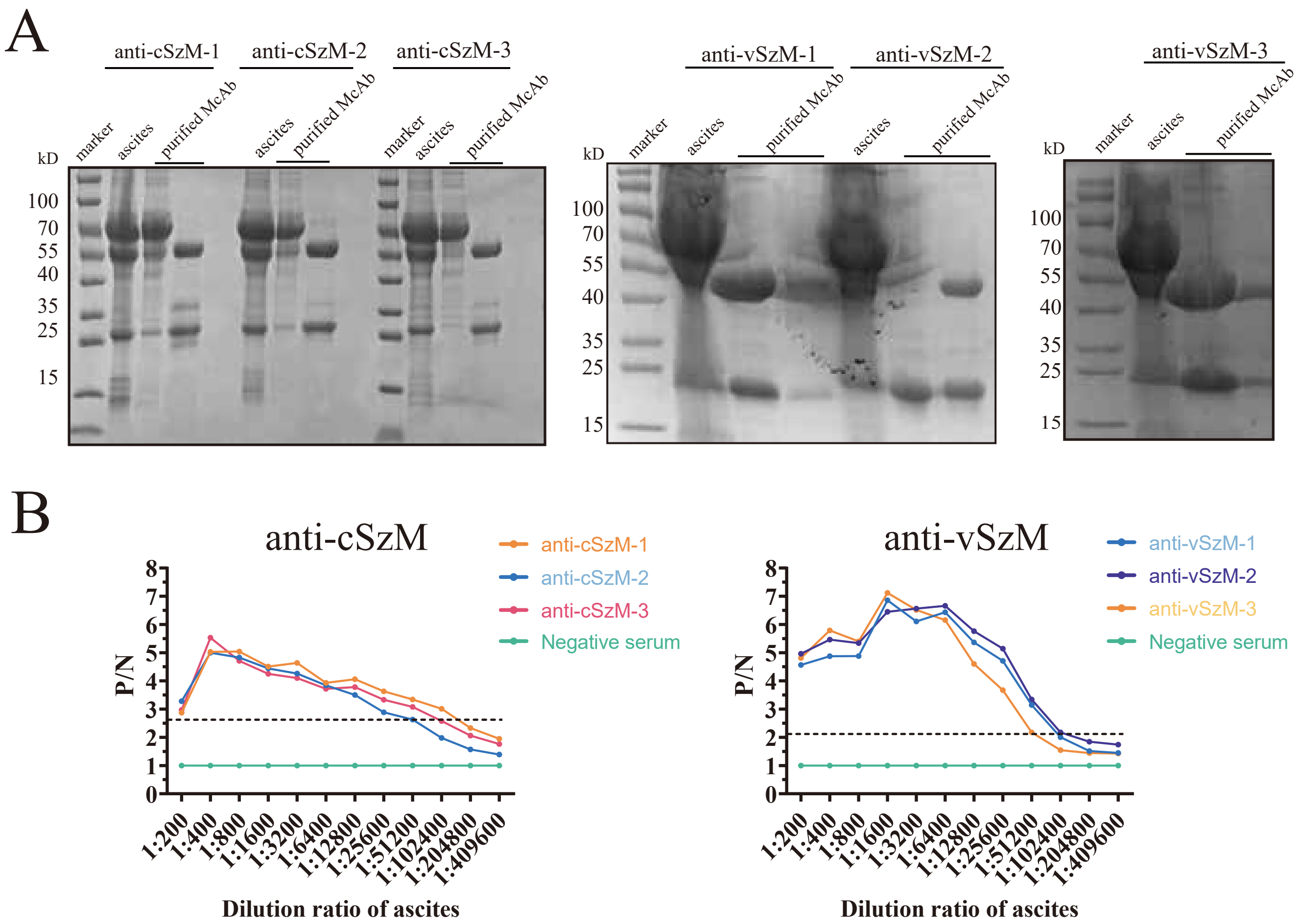

Fig S3

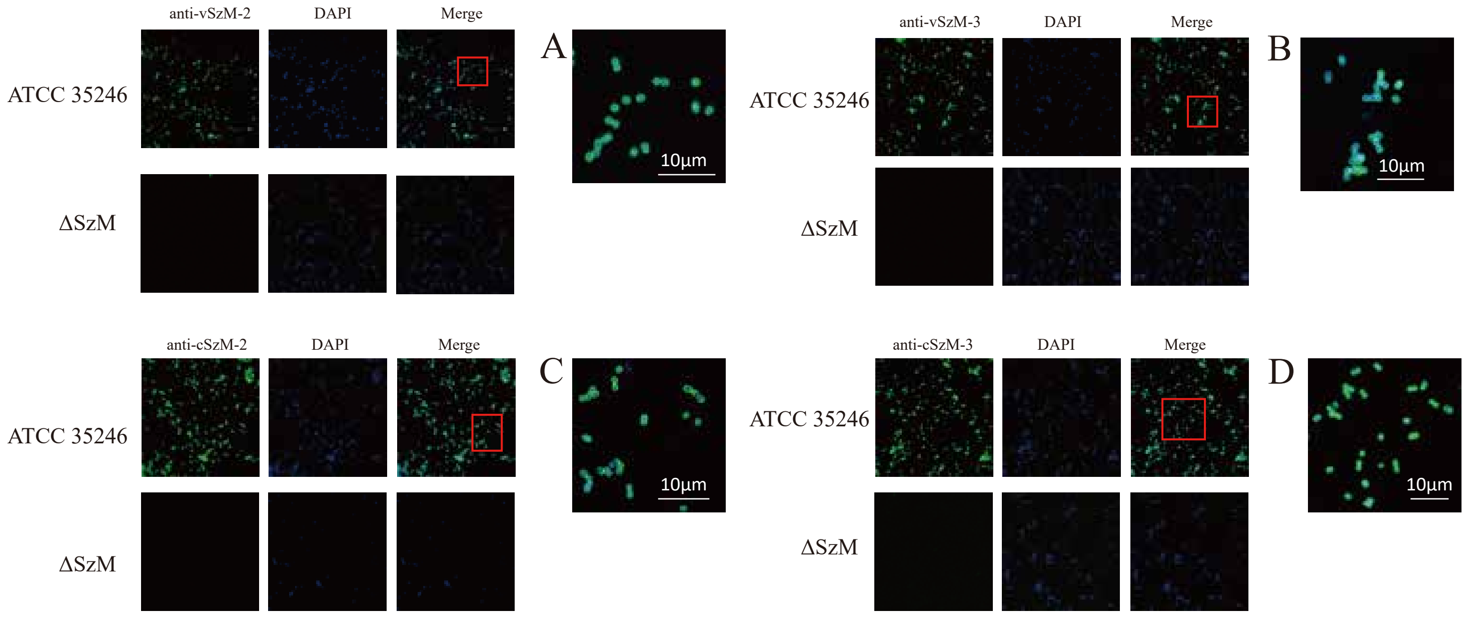

Fig S4

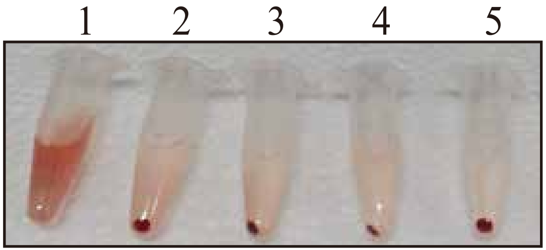

Grouping for complement hemolysis test

Group	Complement	2% Sheep red blood cell	Rabbit anti-sheep erythrocyte antibody
1	√	√	√
2		√	√
3	√（inactivated）	√	√
4	√	√	
5		√	

Fig S5

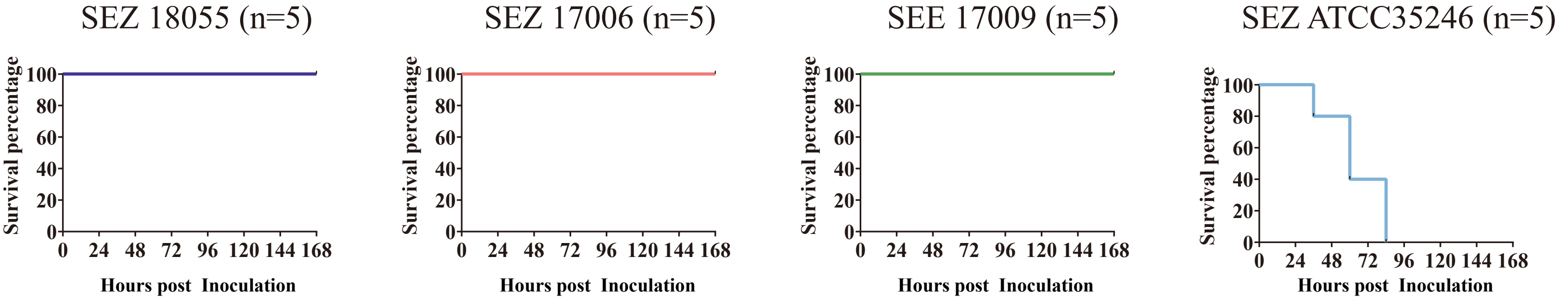

Table S1

Primer name	Primer sequences（5'-3'）
SzM-F	*GTGCGGCCGCAAGCTTTAGTTTTCTTTGCGTTTAGGAGACACC*
SzM-R	*TCGCGGATCCGAATTGTTTTTGAGAAATAACAAGCAAAAATTTAGCATCAGAAAAC*
SzM -truncated-F	*GTGCGGCCGCAAGCTTTGACCAGCTTTAGCAGTTGTAGTGCCT*
SzM-truncated-R	*CGCGGATCCGAATTAGGAGCGGCTGTAAAGGC*
SzM-conserved-F	*GTGCGGCCGCAAGCTTTGACCAGCTTTAGCAGTTGTAGTGCC*
SzM-conserved-R	*TCGCGGATCCGAATTGATCACAACAGAATTAGCTAATAAGTTAA CTGATGCA*
SzM-variant-F	*GTGCGGCCGCAAGCTCTGAATAGCACGGTCTTTATCTTTTTCTG AAGC*
SzM-variant-R	*TCGCGGATCCGAATTAGGAGCGGCTGTAAAGGC*

FIG S1 (A) The diagram of 4 truncated recombinant SzM proteins. SS: Signal peptide sequence; Rep: repeat region; W: LPXTG cell wall insertion motif; tSzM: signal peptide truncated SzM protein; cSzM: conserved region SzM protein; vSzM: variable region SzM protein; rSzM: recombinant whole length SzM protein. The primers used for amplification were marked with green or orange arrow. The relevant sequences of these primers were listed in Table S1. (B) SDS-PAGE of 4 derived recombinant SzM proteins. (C) Identification of 4 recombinant SzM proteins by western blot. The rSzM, tSzM, and cSzM were fused with His tag, and the primary antibody was the anti-His antibody. vSzM was fused with the GST tag, and the primary antibody was the anti-GST antibody.

FIG S2 (A) Six purified ascites antibodies on SDS-PAGE. The heavy chain of antibodies was about 55 kD and the light chain was about 25 kD. (B) Detection of antibody titer in ascites by ELISA. P/N value greater than 2.1 was considered as the positive.

FIG S3 Immunofluorescence assay of ATCC35246 wild-type strain with anti-cSzM-2 and 3, anti-vSzM-2 and 3. The ΔSzM mutant was used as a negative control. The red rectangle area was zoomed in and displayed on the right side.

FIG S4 Complement hemolysis test. When hemolysis occurred, the red blood cells (RBC) were completely ruptured and the supernatant turned to red, which indicated the activation of complement (Tube 1). Tube 2-5 were the negative control which had at least one ingredient absent in the complement system. Details in the right-side table. In the negative control groups, the liquid should turn to transparent and all RBC precipitate in the bottom due to absent of activated complement.

FIG S5 Survival curves of mice challenged with different GCS strains intravenously (10^4 CFU/mouse).

Table S1 Primers' used in this study.
